# Induction of Autophagy in the Striatum and Hypothalamus of Mice after 835 MHz Radiofrequency Exposure

**DOI:** 10.1371/journal.pone.0153308

**Published:** 2016-04-13

**Authors:** Ju Hwan Kim, Yang Hoon Huh, Hak Rim Kim

**Affiliations:** 1 Department of Pharmacology, College of Medicine, Dankook University, Cheonan, Chungnam, Republic of Korea; 2 Nano-Bio EM Research Group, Korea Basic Science Institute, Gwahak-ro, Yuseong-gu, Daejeon, Republic of Korea; IISER-TVM, INDIA

## Abstract

The extensive use of wireless mobile phones and associated communication devices has led to increasing public concern about potential biological health-related effects of the exposure to electromagnetic fields (EMFs). EMFs emitted by a mobile phone have been suggested to influence neuronal functions in the brain and affect behavior. However, the affects and phenotype of EMFs exposure are unclear. We applied radiofrequency (RF) of 835 MHz at a specific absorption rate (SAR) of 4.0 W/kg for 5 hours/day for 4 and 12 weeks to clarify the biological effects on mouse brain. Interestingly, microarray data indicated that a variety of autophagic related genes showed fold-change within small range after 835 MHz RF exposure. qRT-PCR revealed significant up-regulation of the autophagic genes Atg5, LC3A and LC3B in the striatum and hypothalamus after a 12-week RF. In parallel, protein expression of LC3B-II was also increased in both brain regions. Autophagosomes were observed in the striatum and hypothalamus of RF-exposed mice, based on neuronal transmission electron microscopy. Taken together, the results indicate that RF exposure of the brain can induce autophagy in neuronal tissues, providing insight into the protective mechanism or adaptation to RF stress.

## Introduction

The burgeoning use of mobile phones and associated wireless communication devices in the past several decades has led to increasing public concern regarding possible adverse effects of the constant exposure to electromagnetic fields (EMFs) on human health. Compared to the other parts of body, the human brain is exposed to relatively high specific absorption rates (SAR) owing to the close proximity between a mobile phone and the head [1]. A number of animal studies have shown that radiofrequency (RF)-EMFs emitted by a mobile phone results in altered neurotransmitter releases, behavioral change, increased blood-brain barrier (BBB) permeability, increased albumin leak though BBB and neuronal damage in the brain tissues [2–5]. Various neurological and cognitive disorders including dizziness, headaches, visual disruption, sleep disorder, working memory deficits and decreased response times have been reported in epidemiological investigations [6–12]. Certain types of EMF are beneficial to human health, and are used clinically [13–17].

Autophagy is a lysosomal degradative process that involves cellular degradation of unnecessary proteins or damaged organelles by encapsulation in a double-membrane vesicle termed the autophagosome, which then fuses with a lysosome to destroy the engulfed contents [18–20]. Autophagosome formation is mediated by various autophagic genes (ATGs) that regulate initiation, vesicle nucleation, vesicle elongation, autophagosome formation and degradation [18, 21–23].

Autophagy contributes to cellular homeostasis by protecting cells from various environmental stresses and is crucial during cellular differentiation and development [24–26]. However, dysfunction of autophagy is profoundly related to a variety of neurodegenerative disorders due to the aggregation of aberrant proteins due to autophagic defect [27–31]. Low frequency EMF exposure can induce elevated expression of the autophagy related markers Beclin1, Atg7 and LC3-II, and has been associated with the formation of autophagosome dynamics in cultured mammalian cells [32]

The striatum and hypothalamus are important brain tissues that have been linked with neurodegenerative disease and neurological cognitive disorders when mice are constantly exposed to a radiofrequency (RF)-EMF. The striatum is a subcortical part of the forebrain responsible for mediating cognition involving body movement and reward perception [33, 34]. Parkinson's disease results in loss of dopaminergic innervation to the dorsal striatum [35]. Striatum dysfunction is also involved in addiction, bipolar disorder and Huntington's disease [36]. The other brain tissue we focused on this study is the hypothalamus, which is located between the thalamus and the brainstem in the limbic system. The most important function of the hypothalamus is linking the nervous system to the endocrine system [37]. Abnormal functioning of the hypothalamus leads to loss of appetite, changed emotional behavior, infertility, memory loss, lower serotonin and dopamine levels and sleep disorder [38–42].

EMF exposure might induce various diseases or symptoms, but there is very limited information on how EMF affects biological phenotype and symptoms related with brain regions. The present study was performed to determine whether RF exposure could induce autophagy in a rodent model. Differential gene expression in the striatum and hypothalamus was systematically analyzed. Autophagy related genes were affected by RF exposure. Altered gene and protein expression were also studied by using qRT-PCR and Western blotting. Finally, to provide convincing evidence for activation of the autophagic pathway, the fine structure of neurons and whether autophagosomes were formed in the striatum and hypothalamus after RF-EMF exposure were examined by transmission electron microscopy (TEM).

## Materials and Methods

### Animals

Male, 6-week-old C57BL/6 mice weighing 25–30 g were purchased from Daehan Bio Link (Chungbuk, South Korea) and maintained at an ambient temperature of 23 ± 2°C with 12-h light/dark cycles. Food pellets (Daehan Bio Link) and water were supplied *ad libitum*. After a 1-week adaptation period, mice were randomly assigned to groups as described below. All mice procedures adhered to the National Institutes of Health Guidelines for Animal Research and were approved by Dankook University Institutional Animal Care and Use Committee (IACUC; DKU-15-001), which adheres to the guidelines issued by the Institution of Laboratory of Animal Resources.

### RF exposure

Mice were exposed to 835 MHz RF-EMF using a Wave Exposer V20 as previously described [43]. The whole body of each mouse was exposed to 835 MHz radiation at a SAR of 4.0 W/kg for 5 h/day for 4 or 12 weeks. The mice were divided randomly into four groups (n = 5 per group): (i) sham control group for 4 weeks, (ii) 835 MHz RF-exposed group for 4 weeks, (iii) sham control group for 12 weeks and (iv) 835 MHz RF-exposed group for 12 weeks. The sham control groups were kept under the same environmental conditions as the RF-exposed groups but not subjected to RF-EMF exposure. The RF exposed mice move freely in enough space. The space size for mouse cage in RF-EMF generator is approximately 43cm L x 37cm W x 18cm H. This is bigger than standard mouse cage. The RF-EMF exposes from horn antenna to the mouse cage, and bottom and wall of cage is provided by wave absorption material (TDK ceramic absorber) mimicking the radiation exposure in the open environment which excludes the possibility of the influence the number of mice might have on exposure. Importantly, exposure apparatus provides automatic light system and air condition system with water feeder, with no restriction in movement in the cage during the exposure duration eliminating the risk of stress to mice.

### Microarray

Striatal and hypothalamic total RNA extracted from control and RF-exposed mice were analyzed by Nexbio (Deajeon, South Korea) to determine differentially expressed genes (DEGs) by whole genome microarray. Cyanine 3-labeled complementary DNA (cDNA) was generated using a low RNA input linear amplification kit with 500 ng of total RNA and an 8 x 60K SurePrint G3 mouse gene expression hybridization kit (Agilent Technology) was used. DEGs were analyzed using GeneSpring GX 12 (Agilent Technologies).

### RNA extraction and semi-quantitative real-time PCR (sqRT-PCR) and quantitative reverse transcriptase PCR (qRT-PCR)

Total RNA was purified using TRIzol reagent (Thermo Fisher Scientific, Pittsburgh, PA) from the striatum and hypothalamus of mice. RNA was reverse transcribed to cDNA using MMLV reverse transcriptase (Bioneer, Daejeon, South Korea) and an oligo-d(T)18 primer. Semi-quantitative RT-PCR reactions were performed with PCR PreMix (Bioneer). The obtained PCR product of each gene was used for 1.5% agarose gel electrophoresis and the signal intensity of each product was visualized using Syto 60 (Li-COR Biosciences, Lincoln, NB) stained DNA and an Odyssey infrared imaging system (Li-COR Biosciences). qRT-PCR was carried out using a Rotor Gene SYBR Green supermix Kit (QIAgen, Valenica, CA) and fluorescence was measured using a Rotor-gene PCR Cycler (QIAgen). Glyceraldehyde 3-phosphate dehydrogenase (GAPDH) was used as a housekeeping gene. The primers were synthesized from Bioneer. Oligonucleotide sequences of primers [44–46] used for sqRT-PCR and qRT-PCR. The forward and reverse primers were designed as follows: Atg4a F: 5´-CCCTCACACAACCCAGACTT-3´and R: 5´-CCCCTGTGGTTGTCACTTCT-3´; Atg5 F: 5´-GGAGAGAAGAGGAGCCAGGT-3´ and R: 5´-TGTTGCCTCCACTGAACTTG-3´; Beclin1 F: 5´-CTGAAACTGGACACGAGCTTCAAG-3´ and R: 5´-TGTGGTAAGTAATGGAGCTGTGAGTT-3´; LC3A F: 5´-TGGTCAAGATCATCCGGC-3´ and R: 5´-CTCACCATGCTGTGCTGG-3´; LC3B F: 5´-TTCTTCCTCCTGGTGAATGG-3´ and R: 5´-GTGGGTGCCTACGTTCTCAT-3´. GAPDH primer was purchased from QIAgen. Three biologically independent experiments were performed and each PCR reaction was done in triplicate. The relative levels of specific mRNA were calculated by normalizing to the expression of GAPDH by the 2^-ΔΔCt^ method (n = 5). Also, the expression values of the RF-exposed groups were normalized to those of the sham-exposed group.

### Immunoblotting

For Western blot analysis, the hypothalamus and striatum were quickly dissected from mouse brain. Whole striatal and hypothalamic lysates were prepared on ice in RIPA lysis buffer (ATTO, Tokyo, Japan) supplemented with protease inhibitor cocktail (ATTO) and phosphate inhibitor cocktail (ATTO). Whole lysates were homogenized in ice-cold buffer and sonicated for 10 sec. Protein concentrations were estimated using a DC^TM^ protein assay (Bio-Rad, Hercules, CA) and 20 μg of each proteins were subjected to a 15% sodium dodceyl sulfate-polyacrylamide gel electrophoresis and transferred with EzFastBlot transfer buffer (ATTO) to polyvinylidene difluoride (PVDF) membrane (ATTO). Protein bands were visualized using C-DiGit Chemiluminescence Western Blot Scanner (Li-COR Biosciences). Band intensity was quantitated and normalized using α-tubulin as an internal control. In addition, change in the expression level of autophagy related proteins was further analyzed with two-dimensional gel electrophoresis using a PROTEAN i12 IEF System (Bio-Rad).

### TEM

Dissected hypothalamus and striatum were fixed in 2% glutaraldehyde and 2% paraformaldehyde in 0.1 M phosphate buffer (pH 7.4) for 2 h at 4°C. After three washes in phosphate buffer, the tissues were postfixed with 1% osmium tetroxide on ice for 2 h and washed three times, all in phosphate buffer. The tissues were embedded in Epon 812 mixture after dehydration in an ethanol and propylene oxide series. Polymerization was conducted with pure resin at 70°C for 24 h. Ultrathin sections (~70 nm) were obtained with a MT-X ultramicrotome (RMC, Tucson, AZ) and then collected on 100 mesh copper grids. After staining with 2% uranyl acetate for 15 min and with lead citrate for 5 min, the sections were examined by TEM using a Technai G^2^ Spirit Twin microscope (FEI, Hillsboro, OR) operating at 120 kV.

### Statistical analysis

All values are presented as the mean ± SEM with n denoting the number of animals used in experiments. The significance for all pairwise comparisons of interest was assessed by two tailed Student’s *t*-test with probability values of p<0.05 considered significant.

## Results

### Microarray analysis

Microarray analysis was performed to reveal DEGs in the striatum and hypothalamus of mice after exposure to 835 MHz RF radiation at a SAR of 4.0 W/kg for 5 h/day for 4 weeks or 12 weeks. A significant number of genes were differentially affected (data not shown). Expression of autophagy-related genes was altered in both the striatum and hypothalamus (Table 1). Altered expression of Atg4a, Dram1 and Soga1 was apparent after 4 weeks RF exposure, with altered expression of 37 autophagy-related genes in both striatum and hypothalamus evident at 12 weeks (Table 1).

**Table 1:** Microarray analysis of autophagy-related gene expression in the striatum and hypothalamus of mice after 835MHz radiofrequency (RF) exposure for 4 or 12 weeks.

***No*.**	***Gene Symbol***	***Gene Name***	***NCBI accession no*.**	***Fold change (RF vs Control)***	
				*Striatum*	*Hypothalamus*
***4 week RF exposure***					
1	*Atg4a*	autophagy related 4A	NM_174875	1.290	0.066
2	*Dram1*	DNA-damage regulated autophagy modulator 1	NM_027878	-0.242	-0.672
3	*Soga1*	suppressor of glucose, autophagy associated 1	NM_001164663	0.254	-0.620
***12 week RF exposure***					
1	*Ambra1*	autophagy/beclin1 regulator 1	NM_172669	-0.0016	-0.0214
2	*Atg2a*	autophagy related 2A	NM_194348	0.0387	-0.0339
3	*Atg2b*	autophagy related 2B	NM_029654	-0.0845	-0.1751
4	*Atg3*	autophagy related 3	NM_026402	0.2401	0.0717
5	*Atg4a*	autophagy related 4A, cysteine protease	NM_174875	0.0976	-0.0513
6	*Atg4b*	autophagy related 4B, cysteine protease	NM_174874	0.0837	-0.1084
7	*Atg4c*	autophagy related 4C, cysteine protease	NM_175029	-0.0960	0.0233
8	*Atg4d*	autophagy related 4D, cysteine protease	NM_153583	-0.2041	-0.0907
9	*Atg5*	autophagy related 5	NM_053069	-0.0617	0.0270
10	*Atg7*	autophagy related 7	NM_001253717	0.1509	-0.1141
11	*Atg9a*	autophagy related 9A	NM_001288612	-0.3618	-0.1144
12	*Atg9b*	autophagy related 9B	NM_001002897	-0.2310	0.1338
13	*Atg10*	autophagy related 10	NM_025770	0.0402	0.0699
14	*Atg12*	autophagy related 12	NM_026217	-0.1610	-0.0109
15	*Atg13*	autophagy related 13	NM_145528	0.0841	-0.0076
16	*Atg14*	autophagy related 14	NM_172599	-0.1332	0.0932
17	*Atg16l1*	autophagy related 16-like 1	NM_001205392	-0.0091	-0.1176
18	*Atg16l2*	autophagy related 16-like 2	NM_001111111	-0.1649	-0.0933
19	*Atg101*	autophagy related 101	NM_026566	-0.0538	-0.1092
20	*Becn1*	beclin1, autophagy related	NM_019584	0.3314	-0.0429
21	*Dram1*	DNA-damage regulated autophagy modulator 1	NM_027878	-0.5288	1.4058
22	*Dram2*	DNA-damage regulated autophagy modulator 2	NM_026013	0.0979	-0.0915
23	*Epg5*	ectopic P-granules autophagy protein 5 homolog	NM_001195633	-0.0048	-0.0699
24	*Gabarap*	gamma-aminobutyric acid receptor associated protein	XM_006533801	-0.2048	0.0153
25	*Gabarapl1*	gamma-aminobutyric acid (GABA) A receptor-associated protein-like 1	NM_020590	-0.0362	-0.0011
26	*Gabarapl2*	gamma-aminobutyric acid (GABA) A receptor-associated protein-like 2	NM_026693	-0.0316	0.0036
27	*Hdac6*	histone deacetylase 6	NM_010413	-0.0275	0.0318
28	*Irgm1*	immunity-related GTPase family M member 1	NM_008326	0.6942	-0.2102
29	*Lamp1*	lysosomal-associated membrane protein 1	NM_010684	-0.0508	0.0005
30	*Map1lc3a*	microtubule-associated protein 1 light chain 3 alpha	NM_025735	0.0370	0.0225
31	*Map1lc3b*	microtubule-associated protein 1 light chain 3 beta	NM_026160	-0.0225	-0.0634
32	*Npc1*	Niemann-Pick type C1	NM_008720	0.2671	-0.0868
33	*Rgs19*	regulator of G-protein signaling 19	NM_001291206	0.2966	-0.0501
34	*Soga1*	suppressor of glucose, autophagy associated 1	NM_001164663	0.5487	-0.3773
35	*Ulk1*	unc-51 like kinase 1	NM_009469	0.0560	-0.0354
36	*Ulk2*	unc-51 like kinase 2	NM_013881	0.0013	0.1557
37	*Wipi1*	WD repeat domain, phosphoinositide interacting 1	NM_145940	0.2123	-
38	*Wipi1*	WD repeat domain, phosphoinositide interacting 1	AK031672	-	0.1102

### Level of up-regulation of autophagy-related genes in the striatum and hypothalamus after RF exposure

**Fig 1:**
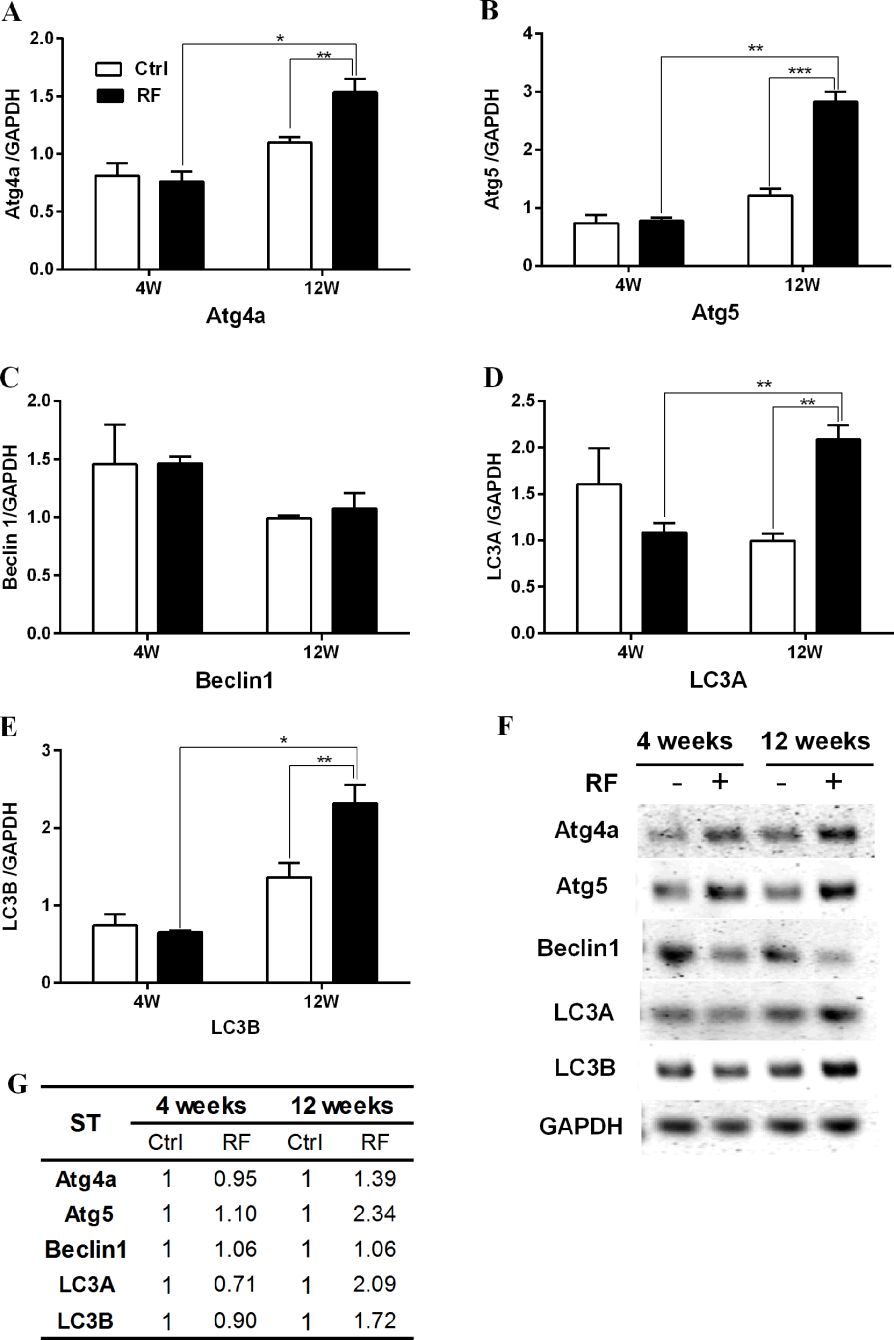
Expression level of autophagy genes in the striatum of mice after 835MHz radiofrequency (RF) exposure. Striatal RNA extracted control and RF-exposed mice were analysed for the expression of autophagy genes by semiquantitative reverse-transcription PCR and quantitative real-time PCR. (A-E) Quantification of Atg4a, Atg5, Beclin1, LC3A and LC3B mRNA by qRT-PCR and (F) Syto60-stained agarose gel showing differential expression of autophagy genes by sqRT-PCR. The expression values of the striatum of RF-exposed mice were normalized to those of the sham-exposed mice. The relative mRNA levels of Atg4a, Atg5, Becn1, LC3A and LC3B was calculated by normalizing to expression of GAPDH by the 2^-ΔΔCt^ method (n = 5). Table (G) shows the average fold-change. Each bar represents the mean ± SEM of three independent experiments. Statistical significance was evaluated using a *t*-test: *P<0.05, **P<0.01, *** P< 0.00.

**Fig 2:**
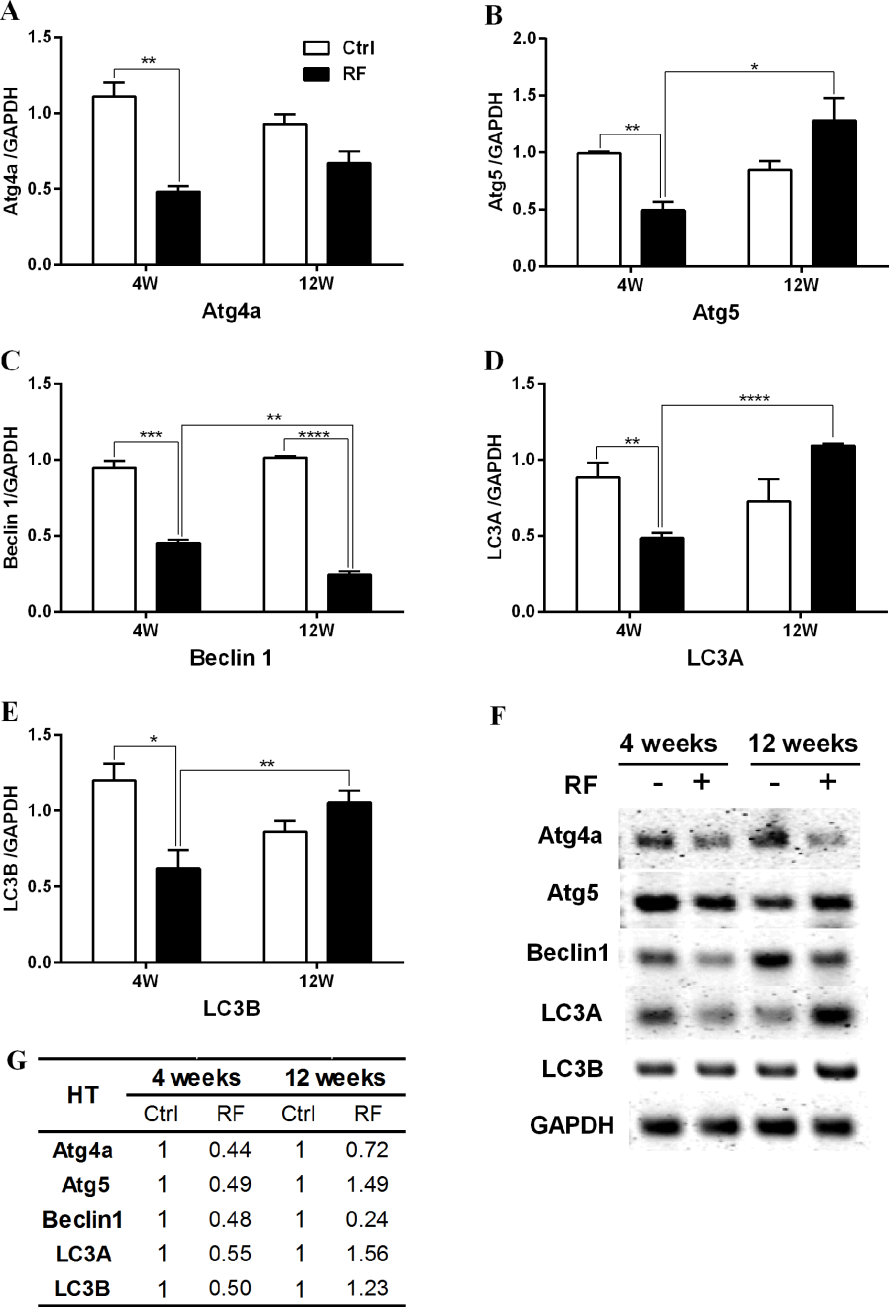
Expression of autophagy genes in the hypothalamus of mice following 835MHz RF exposure. Hypothalamic RNA extracted from control and RF-exposed mice were analysed for the expression of autophagy genes by semiquantitative RT-PCR and quantitative RT-PCR. (A-E) Quantification of Atg4a, Atg5, Beclin1, LC3A and LC3B mRNA by qRT-PCR and (F) expression level of autophagy genes by sqRT-PCR. The expression values of the hypothalamus of RF-exposed mice were normalized to those of the sham-exposed mice. The relative mRNA levels of Atg4a, Atg5, Beclin1, LC3A and LC3B was calculated by normalizing to expression of GAPDH by the 2^-ΔΔCt^ method (n = 5). Table (G) shows the average fold change. Each bar represents the mean ± SEM of three independent experiments. Statistical significance was evaluated using two tailed *t*-test: *P<0.05, **P<0.01, *** P< 0.001, **** P< 0.0001.

To confirm and quantify the altered expression level of autophagy-related genes after RF exposure, specific genes in the striatum and hypothalamus were selected and analysed by both semi-quantitative reverse-transcriptase PCR and qRT-PCR. For RT-PCR, Atg4a, Atg5, Beclin1, LC3A and LC3B were chosen; these genes are crucial for the formation of autophagic vacuoles in response to environmental stress [21]. The qRT-PCR results indicated that 4-week RF exposure changed the expression in the striatum of only LC3A, which was reduced in expression (Fig 1D), while the hypothalamus showed a significant decrease of all five autophagy genes after 4 weeks RF exposure (Fig 2). The expression of Atg4a, Atg5, LC3A and LC3B were significantly increased by about 2-fold following 12 weeks of RF exposure (Fig 1). At that time the hypothalamus also significantly increased levels of Atg5, LC3A and LC3B mRNA (all about 1.5-fold; Fig 2B, 2D and 2E). In parallel, the expression level of Beclin 1 in the hypothalamus was decreased after 4 weeks RF exposure and further diminished up to 0.24-fold after 12 weeks of RF exposure (Fig 2C and 2F). qRT-PCR for Beclin1 in the striatum did not show a significant change of expression (Fig 1C), while sqRT-PCR revealed decreased Beclin 1 mRNA expression in the striatum after 4 and 12 weeks RF exposure (Fig 1F). The rest of the sqRT-PCR and qRT-PCR data were similar. Comparison between the 4 and 12 week control groups revealed unchanged levels of the autophagy-related genes. However, increasing length of RF exposure to mice augmented the expression of Atg4A, Atg5, LC3A and LC3B genes in the striatum, and Atg5, LC3A and LC3B genes in the hypothalamus. These findings suggested that RF-EMF of 4.0 W/kg SAR for 12 weeks was sufficient to activate the autophagy pathway in both the striatum and hypothalamus of mice.

### LC3B-II protein increase in striatum and hypothalamus of mice after 835 MHz RF exposure

**Fig 3:**
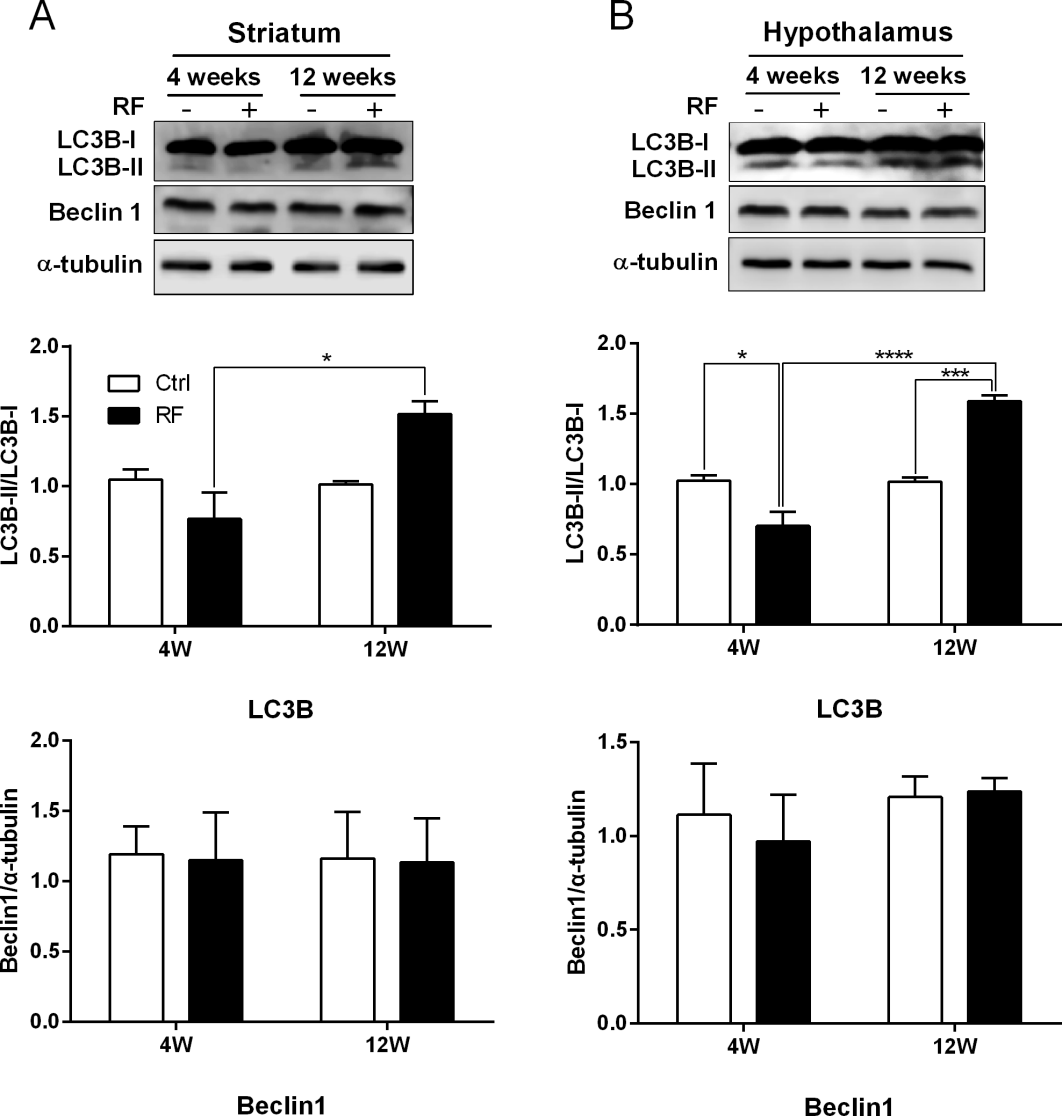
Western blot analysis of autophagy-related proteins in the striatum and hypothalamus of RF-EMF exposed mice. Total protein extracted from mice stratum and hypothalamus was subjected to 15% sodium dodecyl sulfate-polyacrylamide gel electrophoresis and resolved proteins were immunoblotted with antibody against LC3B (A) and Beclin1 (B). The intensity of the bands was quantified by densitometry. The protein level of LC3B-II or Beclin1 was normalized relative to LC3B-I or α-tubulin, respectively. Each bar shows mean of three independent experiments with SEM. Statistical significance was evaluated using two tailed t-test (*P<0.05, *** P< 0.001, **** P< 0.0001).

To validate the results from qRT-PCR and sqRT-PCR, Western blot was used to determine the amount of the relevant proteins produced in the striatum and hypothalamus of mice using anti-LC3B and anti-Beclin 1 antibody. Importantly, the anti-LC3B antibody (Cell Signlaing Technology) detects both mouse LC3B-I (16 kDa) and LC3B-II (14 kDa) protein but maximum concentration of total protein was used for immunoblot in order to detect LC3B-II band. The level of LC3B-II bands were quantified by normalization with LC3B-I. The immunoblot results indicated the pattern of LC3B-II level was quite similar to the gene expression level of LC3B, which showed the high level of LC3B-II expression in both the striatum and hypothalamus of mice exposed to RF for 12 weeks (Fig 3). The data indicated that LC3B-II in both striatum and hypothalamus were significantly increased after 12 weeks RF exposure. However, the expression level of Beclin1 did not change in either brain region (Fig 3).

### Autophagic vacuoles increase in the striatum and hypothalamus of mice after 835 MHz RF-EMF exposure

**Fig 4:**
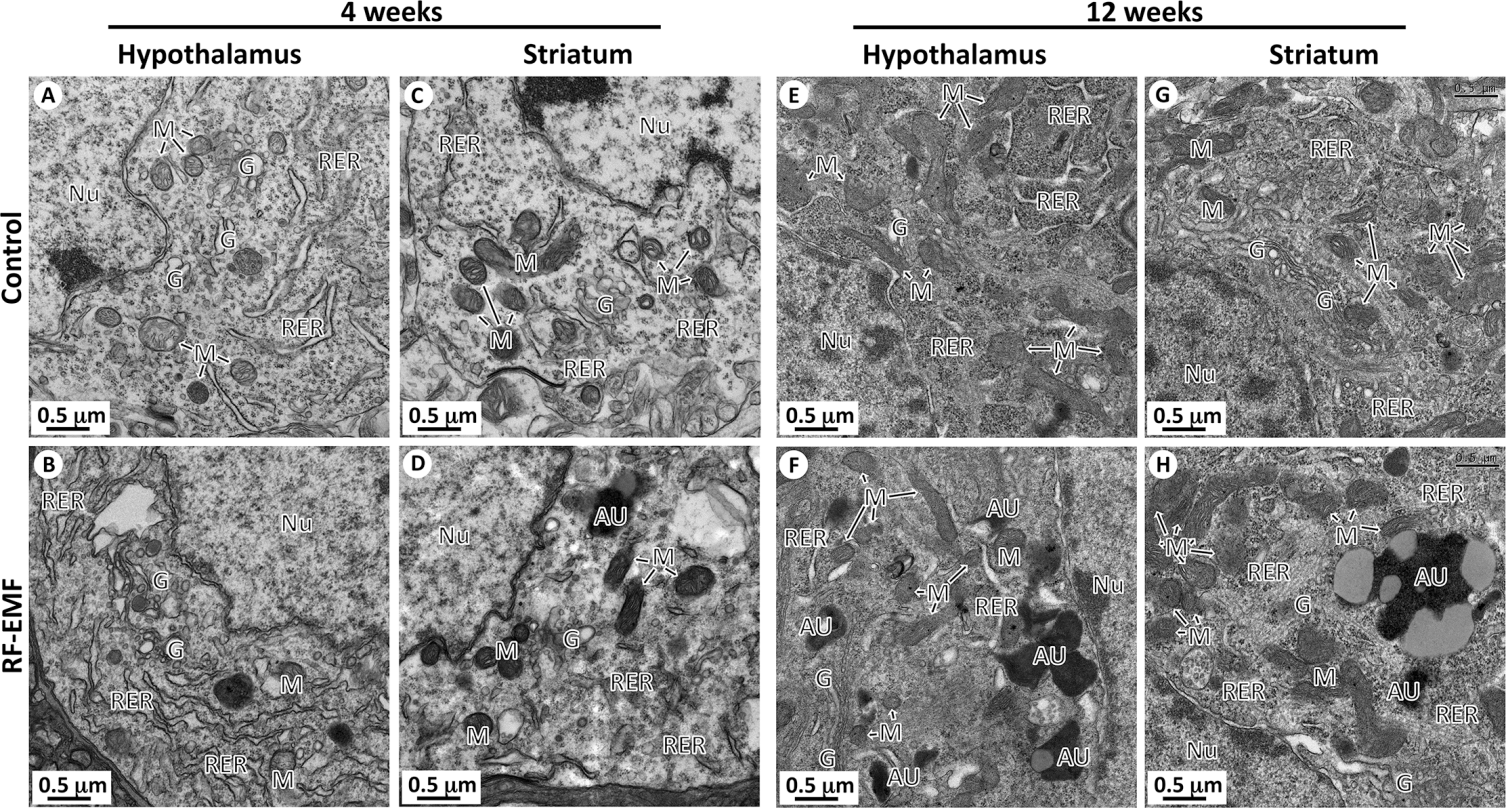
Comparison of intracellular organelles in neuron of hypothalamus and striatum between control and radiofrequency-exposed mice. (A-D). Representative TEM micrographs of neuronal cell body in hypothalamus and striatum were acquired from control mice and radiofrequency-exposed (4 weeks) mice, respectively. Comparative images showing structural indifference of intracellular organelles between control mice and radiofrequency-exposed mice in neuronal cell body of hypothalamus (A vs. B) and striatum (C vs. D). (E-H) Representative TEM micrographs of neuronal cell body in hypothalamus and striatum were acquired from control mice and radiofrequency-exposed (12 weeks) mice, respectively. In radiofrequency-exposed mice during 12 weeks, many autophagy (AU in F and H) were observed in neuronal cell body of hypothalamus (F) and striatum (H) in difference with that of sham control (E and G). However, most of the mitochondria (M), rough endoplasmic reticulum (RER), and Golgi apparatus (Ga) were maintained the structural integrity as similar with that of control mice. Abbreviations are: Nu, nucleus; M, mitochondria; RER, rough endoplasmic reticulum; Ga, Golgi apparatus and AU, autophagy.

Increased expression level of autophagy-related genes and LC3B-II in the striatum and hypothalamus were evident after 12 weeks of RF exposure. Formation of autophagosomes in striatum and hypothalamus neurons was explored using TEM. Autophagic vacuoles were visible in neuronal striatum and hypothalamus after 12 weeks of RF exposure (Fig 4 and S1F and S1H Fig). TEM revealed fewer vacuoles in striatal and hypothalamic neurons after 4 weeks of RF exposure compared to control mice, while the number of autophagic vacuoles was greater and the size of autophagosomes was increased after 12 weeks of RF exposure compared to 12-week control mice (Fig 4). In addition, 12 week RF-exposure to mice observed accumulation of autophagosomes in either brain regions as well as confirmed a number of autolysosome of which these autophagosomes fused to lysosome (S2 Fig). S2 Fig showed the process of autophagosome and autolysosome formation in the striatum of 12 week RF-exposed mice.

However, even with 12 weeks of RF exposure, most of the mitochondria maintained their structural integrity, similar to the non-exposed control mice. Most of mitochondria maintained a dense matrix, were compact with thin and uniform cristae and were surrounded by clear inner and outer membranes (S1 Fig). Intracellular organelles including the nucleus, rough endoplasmic reticulum and Golgi apparatus were similar in control and RF exposed group in neuronal cell bodies of both the striatum and hypothalamus (Fig 4). Overall, these results strongly suggest that 835 MHz RF at 4.0 W/kg SAR for 12 weeks activates the autophagosome formation in the striatum and hypothalamus.

## Discussion

The present findings demonstrate that 835 MHz RF-EMF exposure is involved in the activation of the autophagy pathway in the mouse striatum and the hypothalamus regions of the brain. Because of the proximity between brain and mobile phones, the emitted RF-EMF from mobile phones could be absorbed by the brain and consequently affect brain functions [1]. Numerous EMF studies have failed to quell the debate over the possible deleterious consequences of RF-EMF exposure from mobile phones due to conflicting results of the biological effects of EMF. RF-EMF exposure of the brain could lead to neuronal damage, alter neurotransmitter releases and behavioral change [2, 5]. Epidemiological studies have linked RF-EMF exposure with various neurological and cognitive disorders [6, 8, 9, 12]. RF exposure has also been implicated in apoptosis in cells and an animal model. Exposure of cell to 900 MHz RF produced significant changes in expression levels of genes involved in cell cycle arrest, DNA repair and apoptosis [14, 47–49]. Importantly, similar stimuli can be involved in either autophagy or apoptosis within the same cell, due to possible different sensitivity thresholds that may be determined whether autophagy or apoptosis is triggering [50, 51]. Generally, activation of autophagy blocks the induction of apoptosis by inhibiting activation of Bid or degrading active caspase 8, and activation of apoptosis loses their capacity of the autophagic degradation by which achieves caspase-mediated cleavage of key autophagy proteins such as Beclin 1 or Atg5 [51]. In addition, autophagy is most likely to allow cells to cope with stress but autophagic degradation with large-scale of the cytosol and organelles in the cell would cause cellular dysfunction and eventually lead to cell death [50, 52]. Exposure of rats to 900 MHz RF-EMF significantly increased the number of caspase 3 (a key apoptotic marker)-labeled granule cells and Purkinje neurons in the cerebellum [53]. LF-EMF induced autophagy, based on the increased expression of Beclin1, Atg7 and LC3B-II, in mammalian cells, with the dynamics of autophagic vesicles clarified [32]. However, whether RF-EMF induces neuron autophagy has been unclear.

The present study investigated the biological effects on mouse brain using microarray analysis to study the expression of various genes in mouse striatum and hypothalamus in response to 835 MHz RF (Table 1). More than 30 autophagy-related genes were altered transcriptionally in the 12-week exposure group, with markedly fewer genes affected at 4 weeks exposure. These data were also confirmed through gene expression profile analysis by qRT-PCR and sqRT-PCR (Figs 1 and 2).

Upregulated genes in the striatum and/or hypothalamus in the 12-week RF group were Atg4a, Atg5, LC3A and LC3B (Figs 1 and 2). These genes are pivotal in the formation of autophagic vacuoles [21, 22]. Concerning autophagosome structure, the beclin-1-class III phosphoinositol3kinase complex is necessary for vesicle nucleation and microtubule-associated protein LC3 (light chain 3)-II is inserted into the autophagosome membrane [54]. LC3 is an ubiquitin-like protein that it is cleaved by the cysteine protease Atg4 [55]. LC3-I and phosphatidylethanolamine combine to form LC3-II [56, 57]. This process is required for autophagosome expansion. Atg5 is an E3 ubiquitin ligase-like enzyme that regulates autophagosome elongation by forming a complex with Atg12 and Atg16L1; this complex is required for LC3-I combination to phosphatidylethanolamine to form LC3-II [22, 58]. Presently, RF-EMF exposure significantly increased the protein level of LC3B-II in the striatum and hypothalamus after 12 weeks RF exposure. Localization of LC3B-II to the autophagosomal membrane is a key step in autophagosome formation. Hence the augmented level of LC3B-II might reflect the relative amount of autophagosomes in cells (Fig 3).

However, there is significant depression in the level of autophagic related genes and protein expression in hypothalamus at the 4 weeks RF exposure. It is of interest that the regulation of autophagy is different depending on the period of RF exposure. The 835 MHz RF exposure at 4.0 W/kg may act as a big stress to mice brain at the early periods, thus, this stimulus triggers a programmed cell death (apoptosis) in hypothalamus, which induced down-regulating the expression level of autophagy related genes at 4 weeks [50, 51]. To identify the change of morphology in mouse brain neurons after RF exposure, the striatal and hypothalamic neurons were examined by TEM. As we expected based on the gene and protein expression data, autophagic vacuoles were evident in the neuronal striatum and hypothalamus after 12 weeks of RF exposure (Fig 4 and S1F and S1H Fig). Striatal and hypothalamic neurons examined after 4 weeks of RF exposure contained a small number of vacuoles relative to control. But, a number of autophagosomes were observed at 12 weeks relative to the control group (Fig 4). 12 week RF-exposure to mice confirmed a number of autolysosome of which these autophagosomes fused to lysosome and further showed the process of autophagosome that are engulfed cell materials or protein aggregates and autolysosome formation in the striatum (S2 Fig). However, even after 12 weeks of RF exposure, no changes were apparent in the structure of intracellular organelles including mitochondria, nucleus, rough endoplasmic reticulum and Golgi apparatus in both the striatum and hypothalamus (Fig 4 and S1 Fig). These results strongly support activated formation of autophagosomes in both the striatum and hypothalamus by 835 MHz RF at 4.0W/kg SAR exposure for 12 weeks. This result is meaningful because exposure of RF-EMF could induce autophagy, which may maintain neuronal homeostasis and help maintain normal brain function. Autophagy is a survival mechanism that involves inhibited accumulation of abnormal proteins, which can disrupt neural function and lead to neurodegenerative disorders [27, 28, 30]. Moreover, RF-EMF exposure could not induce neuronal cell damage in our system because the stress levels from 835 MHz RF might be just sufficient enough for induction of autophagy but not induce the neuronal impairment.

Our results provide support for the hypothesis that autophagy can be triggered in the brain under RF stress as a means of neuronal protection. Basically, the striatum helps body movement and striatal dopamine neurons are important for dopamine transporter. The hypothalamus controls hormones and further regulates cognition. So, dysfunction of striatum or hypothalamus could fuel neurodegenerative or cognitive disorders, respectively [33, 34, 36, 38, 42]. Hence, autophagy response to RF exposure might help avoid or delay neuronal cell death, which underlie neurological or neurodegenerative diseases, and further protect the neurons. Induction of autophagy in brain tissues during the 12-week exposure to 835 MHz RF at 4.0W/kg may have provided an appropriate stress environment to trigger the cellular survival mechanism. The RF exposure may also have contributed to organelle injury and protein aggregation, and adaptation to RF stress through the autophagy mechanism. No distinctive differences of behaviors or particular phenotypes between control and RF-exposed mice were evident throughout the experimental period.

Although RF-EMF induces the autophagic pathway, the effects of EMF on the brain still remain unclear. Here, we focused on the striatum and hypothalamus that are located in the interior of the brain. Other regions need to be studied including the cerebral cortex located outside of the brain, as well as brain tissues like the brainstem and hippocampus.

In summary, autophagy can be induced in striatum and hypothalamus after exposure of C57BL/6 mice to 835 MHz RF-EMF at a SAR of 4.0W/kg for 5h/day for 12 weeks, with increased expression of autophagic genes and protein (e.g., LC3B-II) being evident. Autophagosomes form in neuronal cell bodies of the hypothalamus and striatum regions. These results may provide insight into the adaptation to stress and further protective mechanism by the autophagic accumulation in neurons against the stress induced by RF-EMF exposure, which might be important in maintaining normal brain functions and maintaining normal behavior.

## Supporting Information

S1 Fig Analysis of mitochondria structure in neuron of hypothalamus and striatum between control- and radiofrequency-exposed mice. (A-D) Representative TEM micrographs of mitochondria in neuronal cell body of hypothalamus (A and B) and striatum (C and D) acquired from 4 week radiofrequency-exposed mice and age-matched control mice. (E-H) Representative TEM micrographs of mitochondria in neuronal cell body of hypothalamus (E and F) and striatum (G and H) acquired from 12 week radiofrequency-exposed mice and age-matched control mice. Even the radiofrequency exposure for 12 weeks, most of the mitochondria (M) were maintained the structural integrity as similar with that of control mice. They were maintained a dense matrix, compacted with thin and uniform cristae, and surrounded by clear inner and outer membrane. Abbreviations are: Nu, nucleus; M, mitochondria; RER, rough endoplasmic reticulum; Ga, Golgi apparatus and AU, autophagy.
(TIF)

S2 Fig A series of representative TEM images showing the presumed progressing of the autophagosomic-lysosomal degradation in the striatum of mice following three month RF-EMF exposure. (A-J) Low power images of the process of autophagy. (A’-J’) High power images of the each insert (black dotted box) in A-J, respectively. (A’) Condensed thick phagophore. (B’ and C’) Phagophore (D’) early autophagosome containing fragment of cytoplasmic organelles. (E’) Autophagosome. (F’) Autophagosome with adjacent lysosome. (G’-J’) Autolysosomes. Ph, phagophore; M, mitochondria; Ly, lysosome; Arrow, autophagosome; Arrowhead, autolysosome. Size bars: 500 nm.
(TIF)
