# Supplementary figures and images for: Induction of Autophagy in the Striatum and Hypothalamus of Mice after 835 MHz Radiofrequency Exposure
Source: PLoS One. 2016 Apr 13;11(4):e0153308. doi: 10.1371/journal.pone.0153308 (PMC4830612; doi:10.1371/journal.pone.0153308)

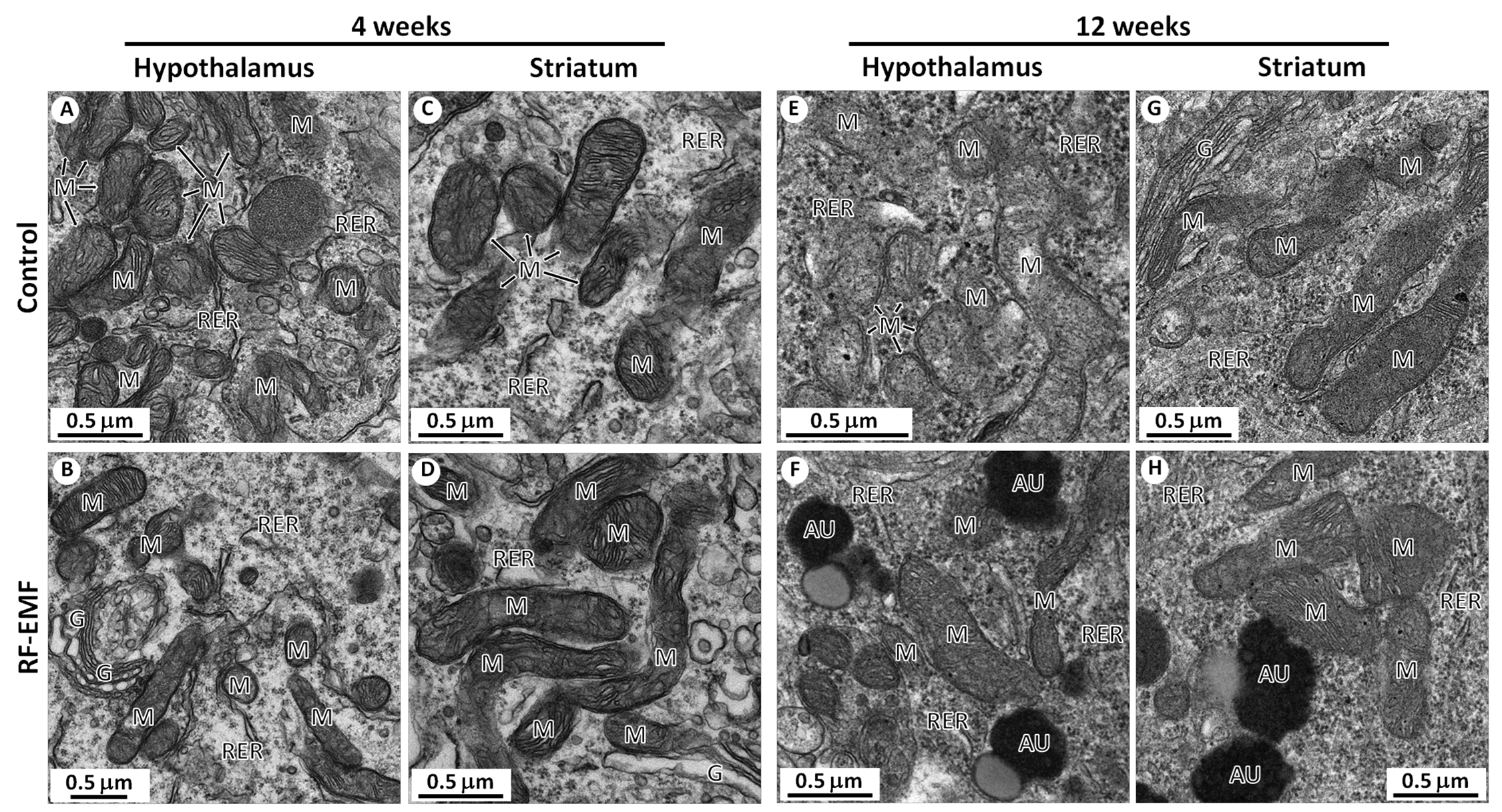

Supplement: S1 Fig — (A-D) Representative TEM micrographs of mitochondria in neuronal cell body of hypothalamus (A and B) and striatum (C and D) acquired from 4 week radiofrequency-exposed mice and age-matched control mice. (E-H) Representative TEM micrographs of mitochondria in neuronal cell body of hypothalamus (E and F) and striatum (G and H) acquired from 12 week radiofrequency-exposed mice and age-matched control mice. Even the radiofrequency exposure for 12 weeks, most of the mitochondria (M) were maintained the structural integrity as similar with that of control mice. They were maintained a dense matrix, compacted with thin and uniform cristae, and surrounded by clear inner and outer membrane. Abbreviations are: Nu, nucleus; M, mitochondria; RER, rough endoplasmic reticulum; Ga, Golgi apparatus and AU, autophagy. (TIF) [file pone.0153308.s001.tif]

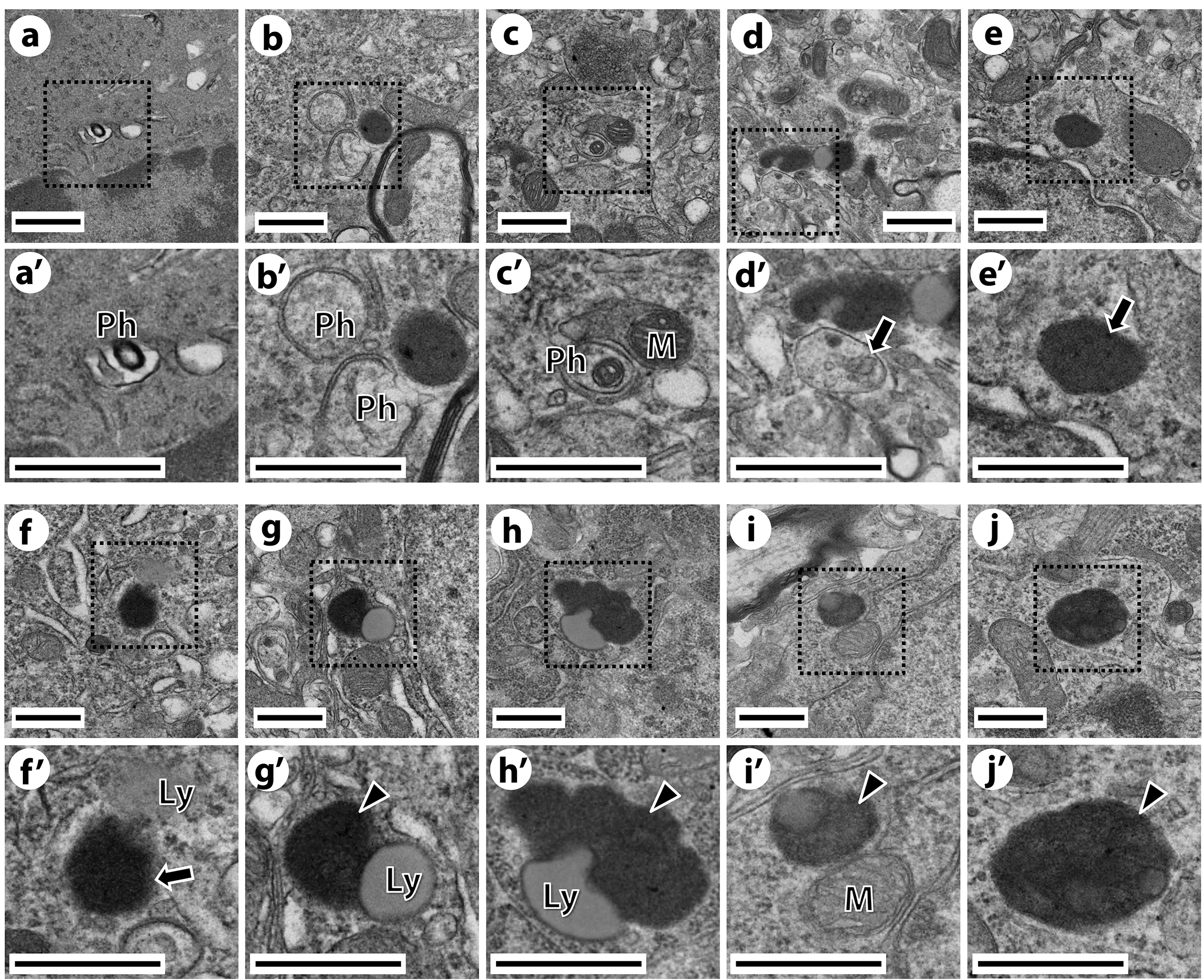

Supplement: S2 Fig — (A-J) Low power images of the process of autophagy. (A’-J’) High power images of the each insert (black dotted box) in A-J, respectively. (A’) Condensed thick phagophore. (B’ and C’) Phagophore (D’) early autophagosome containing fragment of cytoplasmic organelles. (E’) Autophagosome. (F’) Autophagosome with adjacent lysosome. (G’-J’) Autolysosomes. Ph, phagophore; M, mitochondria; Ly, lysosome; Arrow, autophagosome; Arrowhead, autolysosome. Size bars: 500 nm. (TIF) [file pone.0153308.s002.tif]
